# Measuring the hidden burden of violence: use of explicit and proxy codes in Minnesota injury hospitalizations, 2004–2014

**DOI:** 10.1186/s40621-021-00354-6

**Published:** 2021-11-01

**Authors:** N. Jeanie Santaularia, Marizen R. Ramirez, Theresa L. Osypuk, Susan M. Mason

**Affiliations:** 1 Division of Epidemiology and Community Health, University of Minnesota School of Public Health, 300 West Bank Office Building, 1300 S. 2nd St., Minneapolis, MN 55454 USA; 2 Minnesota Population Center, University of Minnesota, 225 19th Ave S #50th, Minneapolis, MN 55455 USA; 3 Division of Environmental Health Sciences, University of Minnesota School of Public Health, 1260 Mayo Building, MMC 807, 420 Delaware St. SE, Minneapolis, MN 55455 USA

**Keywords:** Violent injury, Surveillance, Hospital data, Child abuse, Intimate partner violence, Elder abuse

## Abstract

**Purpose:**

Commonly-used violence surveillance systems are biased towards certain populations due to overreporting or over-scrutinized. Hospital discharge data may offer a more representative view of violence, through use of proxy codes, i.e. diagnosis of injuries correlated with violence. The goals of this paper are to compare the trends in violence in Minnesota, and associations of county-level demographic characteristics with violence rates, measured through explicitly diagnosed violence and proxy codes. It is an exploration of how certain sub-populations are overrepresented in traditional surveillance systems.

**Methods:**

Using Minnesota hospital discharge data linked with census data from 2004 to 2014, this study examined the distribution and time trends of explicit, proxy, and combined (proxy and explicit) codes for child abuse, intimate partner violence (IPV), and elder abuse. The associations between county-level risk factors (e.g., poverty) and county violence rates were estimated using negative binomial regression models with generalized estimation equations to account for clustering over time.

**Results:**

The main finding was that the patterns of county-level violence differed depending on whether one used explicit or proxy codes. In particular, explicit codes suggested that child abuse and IPV trends were flat or decreased slightly from 2004 to 2014, while proxy codes suggested the opposite. Elder abuse increased during this timeframe for both explicit and proxy codes, but more dramatically when using proxy codes. In regard to the associations between county level characteristics and each violence subtype, previously identified county-level risk factors were more strongly related to explicitly-identified violence than to proxy-identified violence. Given the larger number of proxy-identified cases as compared with explicit-identified violence cases, the trends and associations of combined codes align more closely with proxy codes, especially for elder abuse and IPV.

**Conclusions:**

Violence surveillance utilizing hospital discharge data, and particularly proxy codes, may add important information that traditional surveillance misses. Most importantly, explicit and proxy codes indicate different associations with county sociodemographic characteristics. Future research should examine hospital discharge data for violence identification to validate proxy codes that can be utilized to help to identify the hidden burden of violence.

**Supplementary Information:**

The online version contains supplementary material available at 10.1186/s40621-021-00354-6.

## Introduction

Violence is a common and serious public health problem. In 2019, there were an estimated 5.4 million violent victimization experiences among U.S. residents 12 or older (Morgan and Jennifer 2020). In order to track incidence, accurate surveillance of violent events is critical. Administrative data for violence identification typically occur in two systems: first, systems where the primary purpose is violence identification, and, second, systems where the primary purpose is not violence identification. An example of the former is child protective services (CPS) data, where the primary function is to identify children who are victims of violence and related victimization (e.g., neglect). These and other types of administrative systems designed to capture violence cases, such as police data, are most commonly used for violence surveillance. However, there has been concern about bias in these data, because they only capture a fraction of cases, due to under-reporting (Rochelle and Buonanno 2018; Gray et al. 2017; Lipsky et al. 2012; Feldman et al. 2017). Further, the cases that are captured may skew toward more highly scrutinized communities (e.g., those with interaction with mandated reporters through public benefits programs) (Maguire-Jack et al. 2018).

Because of concerns with administrative data designed for violence identification, there has been increasing attention to the utility of administrative data where the primary function is not violence identification. An example of this second type of administrative data is hospital claims databases, where the primary function is hospital billing. These databases are formed using medical records. The medical records are used by health information professionals to code a patient's visit for billing purposes, using three main types of codes: International Classification of Disease Clinical Modification, 9th Revision (ICD-9 CM codes or hereafter ICD-9 codes), external cause (E-codes), and supplementary classification factors influencing health status codes); since October 2015, billing coding is based on the ICD-10-CM. ICD-9 CM codes are primarily used to assess cost and reimbursement, but they are widely available and therefore are also useful in surveillance and research on disease morbidity and mortality (Thomas et al. 2002), and may be similarly used to study violence (Ahern et al. 2018).

A common way to identify violence in hospital billing data is through ICD codes that explicitly diagnose an injury as caused by violence (e.g., ICD-9 CM: 995.81 for physical abuse) (Scott et al. 2009). However, these so-called “explicit” codes are underutilized (Muldoon et al. 2019) and may be biased in similar ways to police or child protection data. For example, for an ICD violence code to be assigned, either the patient must reveal that the injury that brought them into the hospital was due to violence, or the provider must make a subjective assessment that the intent of the injury was violence since the coding system does not allow for suspected abuse or neglect to be documented. There are several reasons why patients may not disclose violence, including presence of the perpetrator in the hospital room with the patient/victim (Lipsky et al. 2009; Hymel et al. 2018; Lane et al. 2002). Other sources of bias as it relates to violence codes could be related to the patients socio-economic status (Keenan et al. 2017), resource barriers (Beynon et al. 2012), and urban versus rural setting (Edwards 2015). More specifically, these codes could be overutilized for patients of color or low socioeconomic status individuals and underutilized for rural communities where violence tends to be underreported. This then would bias associations when examining how these factors are associated with violence. Thus, cases identified through these explicit codes may not be representative of the true distribution of violence-related injuries (Scott et al. 2009; Schnitzer et al. 2011; Lloyd and Rissing 1985; McKenzie et al. 2011; Hooft et al. 2015).

A second option for assessing violence in hospital data is through “proxy” codes for injuries. Proxy codes are common outcomes of violence (Schnitzer et al. 2011; Bhargava et al. 2011). Using these “proxy” codes for violence identification may yield a more representative distribution of violence and less prone to bias because it does not rely on patient reports or subjective assessments by providers. Past research has examined the correlation of these proxy codes with “gold standard” violence identification methods. For example, in child maltreatment, one study (Schnitzer et al. 2011) used medical record review by project staff trained in child maltreatment, as well as consultation by an advisory board, to identify maltreatment cases. An ICD code was defined as “suggestive of child maltreatment” when greater than 66% of the visits with that code were thought to be caused by child maltreatment (Schnitzer et al. 2011). An example of these newly identified proxy codes included retinal hemorrhage. Intimate partner violence (IPV) proxy codes have been identified through a confluence of studies linking head, neck and face injuries to IPV (Bhargava et al. 2011; Perciaccante et al. 1999, 2010; Schafer et al. 2008; Davidov et al. 2015; Wu et al. 2010; Petridou et al. 2002; Bhandari et al. 2006; Halpern et al. 2009; Sheridan and Nash 2007). Specifically, one study used predictive models to examine which injuries correctly predicted IPV cases confirmed through telephone or clinical diagnoses (Bhargava et al. 2011). Another study found that head, neck and face injuries had a 91% sensitivity and 59% specificity for violence against women (Perciaccante et al. 1999). It is important to note that, high specificity means low type 1 error (low likelihood of finding false positive) but low specificity does introduce potentially type 2 error (false negative). Lastly, elder abuse proxy codes have been identified through common diagnoses among individuals who reported to Adult Protective Services (APS) (Wiglesworth et al. 2009). These methodologies (and others, Lachs et al. 1997; Gironda et al. 2016) provide an evidence base for use of proxy ICD codes to identify violent injury.

These two options of using explicit vs proxy ICD hospital codes for violence identification have trade-offs. For instance, explicit codes are very likely to reflect true violence, but are potentially biased. In contrast, proxy codes are likely to include injuries not due to violence, but this misclassification is more likely to be non-differential and may result in less systematic bias. While proxy codes are not currently used for violence surveillance, they could potentially address the impacts of systematic underreporting, improving case ascertainment, and thus reveal the hidden burden of violence. To move toward the use of proxy codes for surveillance, more needs to be understood about how proxy codes compare and contrast to explicit codes with regard to their temporal and geographic distribution. However, there is no research, to our knowledge, that compares proxy codes to explicit codes in terms of trends over time, and association with county-level predictors of violence such as poverty, racial/ethnic demographics, urbanicity, employment, and education level. The contribution of such a comparison adds important information on potentially differential patterns especially in specific subgroups that are over-scrutinized and those where violence tend to be culturally silent (e.g., rural, and elder abuse).

The goals of this paper are therefore to compare the trends in violence in Minnesota by county from 2004 to 2014, and associations of county-level demographic characteristics with violence rates as measured through explicit, proxy and a combination of explicit and proxy codes using Minnesota Hospital Discharge Data. Three violence subtypes (child maltreatment, elder abuse, and intimate partner abuse) are examined to represent three important types of violence over the lifetime.

## Methods

### Data

#### Minnesota hospital discharge data

Population representative hospital administrative data from 2004 to 2014 were obtained through the Minnesota Hospital Association (MHA). Minnesota hospitals (*n* = 246) are required to submit all inpatient, outpatient, and emergency department claims data to MHA, which compiles these data in a statewide administrative claims database. This database contains a data point for each patient encounter with a health care provider, including diagnoses (ICD codes). Individuals could appear in the database multiple times.

ICD-9 CM codes are used to describe the diagnosis of the condition being treated. For injuries, they describe the nature of the injury (e.g. facture, cut, etc.) and body part (skull, arm, etc.). ICD-9 CM codes are the main codes included in administrative datasets because they are required for billing and reimbursement. External cause codes (E-codes) are optional additional descriptors to ICD-9 CM codes that describe when and where the injury happened, to whom or by whom, how, and intentionality (Injury Data and Resources 2015). V-codes are supplementary classification of factors influencing health status. Cross-sectional (not longitudinally-linked) MHA data on ICD-9 CM, E-codes, and V-codes from 2004 to 2014 were used to measure cases of violence for this study. These data become the numerator for the violence rates.

#### Population data

Population denominators to calculate county-level violence-related injury rates were obtained from the Surveillance, Epidemiology, and End Results (SEER) Program (National Cancer Institute 2021) which provides annual population level estimates by county, sex, and age for each year from 2004 to 2014. The following denominators were used for each violence subtype: age 0 to 18 for child abuse, 65 plus for elder abuse, and 16 plus for intimate partner violence.

#### Sociodemographic data

The 2010 Decennial Census (US Census Bureau 2021a) and the 2010 American Community Survey (ACS) (US Census Bureau 2021b) were used to provide county-level sociodemographic predictors (correlated with violence in traditional systems) of county-level violence (United States Census Bureau 2020) including: percent poverty, percent minority, urban, percent unemployed and percent less than high school education. The continuous variables are dichotomized at the mean.

### Case ascertainment

Violence-related injuries were identified using both explicit and proxy methods. To avoid duplication, using a unique encounter-specific identifier, encounters with both an explicit and a proxy code were identified in the explicit count. Three subtypes of violence were analyzed: child maltreatment (people ages 0–17 years), elder abuse (people ages 65+ years) and intimate partner violence (people ages 16+ years). Appropriate population denominators were applied to create incidence rates. These three subtypes of violence were chosen based on being representative of different forms of violence throughout one’s lifespan and availability of research identifying proxy codes, as described below.

#### Explicit operationalization of violence

ICD-9 CM codes, E-codes, and V-codes that indicate a diagnosis of violence (explicit codes) are listed in Table 1 along with corresponding average yearly counts.

**Table 1:** Explicit and proxy ICD-9, E- and V-codes used to define violence in minnesota hospital discharge data

	Explicit code(s)	*n*^a^		Proxy code(s)^b^	Exclusion code(s)^c^	Age restriction	Sex	*n*^a^
*Child abuse*								
Abuse	995(.50–.55, 0.59)	315	Sexual abuse related codes	54.1, 98, 614.9, 922.4, V71.5, V71.81,	NA	< 10	NA	855
Abandonment	E904.0, E968.4	14	Neglect related codes	692.7	NA	< 2	NA	0
Abuse by varies perpetrators	E 967.0–.9	260		994.1, E910(.2,.4,.8,.9), E960.0	NA	< 4	NA	119
Assault/homicide (struck by or against)	E960.0, E968.2	1474		808, 860, 861, 863.8, 864,866,941,942,945,946, 960–979, E980	NA	< 5	NA	0
Assault/homicide (fire or burn)	E961, E965.6, E968(.0, .3)	4		521, 262, E869.4, E985, V60	NA	< 10	NA	154
Assault/homicide (poisoning)	E962(.0–.2,.9)	7	Neglect or physical abuse related codes	952	NA	< 3	NA	0
Assault/homicide (suffocation)	E963	7		800, 805, 852, 862, 863.2, 863.3, 865	NA	< 5	NA	0
Assault/homicide (cut)	E966	142	Physical abuse related codes	362.81	NA	< 3	NA	4
Assault/homicide (drowning)	E964	1		E965, E966, E968.2, E968.9	NA	< 4	NA	15
Assault/homicide (firearm)	E965(.0–.4)	98		807.0, 807.1, 811, 852.2, 853	NA	< 5	NA	0
Assault/homicide (fall)	E968.1	3		863.1, E988	NA	< 10	NA	0
Assault/homicide (vehicle)	E968.5	4						
Assault/homicide(other)	E965(.5–.9), E967(.0–.9), E968 (.4,.8,.9), E969	637						
*Elder Abuse*								
Abuse	995(.80-.85)	5	Abrasion	910–919	E928.9	≥ 65	NA	0
Abuse by varies perpetrators	E 967.0-.9	27	Bruise	920–924	E928.9	≥ 65	NA	4500
Assault/homicide (struck by or against)	E960.0, E968.2	22	Burns	940–949	E928.9	≥ 65	NA	0
Assault/homicide (fire or burn)	E961, E965.6, E968(.0, .3)	90	Dehydration	276.51	E928.9	≥ 65	NA	8716
Assault/homicide (poisoning)	E962(.0–.2,.9)	1	Laceration	870–897	E928.9	≥ 65	NA	0
Assault/homicide (suffocation)	E963	0	Malnutrition	262–263	E928.9	≥ 65	NA	10
Assault/homicide (cut)	E966	1	Pressure ulcer	707	E928.9	≥ 65	NA	0
Assault/homicide (drowning)	E964	9	Strangulation	E963	E928.9	≥ 65	NA	1
Assault/homicide (firearm)	E965(.0–.4)	0						
Assault/homicide (fall)	E968.1	2						
Assault/homicide (vehicle)	E968.5	1						
Assault/homicide(other)	E965(.5–.9), E967(.0–.9), E968(.4,.8,.9), E969	1						
Rape	E960.1	90						
*Intimate partner violence*								
Abuse by ex-partner, ex-spouse, spouse, partner	E967.3, V611.1	653	Injuries to face/head/neck	802.0–802.9, 873.0–873.9,900.0–900.9, 910(.0–.3, .6–.9), 918.9, 920.0–921.9, 940.0–940.5, 940.9, 941.00–941.59, 959.01, 959.09	E810.0-E819.9	≥ 16	Female	44,435

^a^Average yearly count. Does not sum to sample size because there could be multiple diagnoses per individual

^b^Details in the Additional file 1: Appendix Table 1

^c^Codes used in combination with proxy codes that would exclude the count of a proxy code if the record also had the exclusion code

#### Proxy operationalization of violence

ICD-9, E-Codes, and V-codes indicating injury suggestive of violence (proxy codes) are listed in Table 1 along with corresponding average yearly counts. The proxy operationalizations here were based on a review of literature. The final selection of proxy codes were based on studies identifying these codes through confirmed violence cases via in-depth medical record review (Schnitzer et al. 2011; Gironda et al. 2016; Btoush et al. 2009; Barlow et al. 1998), predictive modeling (Bhargava et al. 2011; Perciaccante et al. 1999, 2010; Reis et al. 2009), common diagnoses of known violent encounters (Schafer et al. 2008; Davidov et al. 2015; Wu et al. 2010; Petridou et al. 2002; Bhandari et al. 2006; Halpern et al. 2009; Sheridan and Nash 2007; Wiglesworth et al. 2009; Nannini et al. 2008; Rosen et al. 2016) and linking hospital records with known cases of violence through administrative data systems (Schnitzer et al. 2011; Lachs et al. 1997) such as Child Protection Services (CPS) or Elder Protection Services. Codes from these studies were only selected if there is consistency across literature for certain types of injuries and/or most likely to be violence, as indicated by the study. Motor vehicle crashes was excluded from IPV to minimize non-violence-related injuries (Sheridan and Nash 2007). Significantly less literature was available on elder abuse. Therefore, to increase certainty in elder abuse proxy codes, injuries identified as being “unintentional intent” e.g., (E928.9) were excluded from elder abuse. The resulting elder abuse proxy codes were therefore restricted to either undetermined or intentional injuries. Further descriptions of the proxy codes are in Additional file 1: Appendix Table 1.

#### Combined operationalization of violence

The counts for the number of explicit and proxy operationalization of violence codes were summed to create a third operationalization of violence. The combined outcome was meant to serve as an intermediary approach for identifying violence.

### Analysis

The distribution and time trends of explicit and proxy child abuse, IPV, and elder abuse by all 87 counties in Minnesota from 2004 to 2014 were examined. For incidence rates, the yearly sum of cases in a county, defined by the given set of codes, served as the numerator and yearly county population data served as the denominator.

To estimate associations between county-level risk factors (e.g., poverty) and county violence rates, negative binomial regression models with generalized estimation equations were run to estimate incidence rate ratios with 95% CIs, accounting for within-county clustering over time. Two separate models were run for each outcome, crude and adjusted. First, crude models with the yearly count totals of each outcome were regressed separately on each individual socio-demographic variable and on year, with the yearly county-level population denominator for the offset (rate denominator). Second, fully-adjusted models that included all the county-level socio-demographic variables and year were estimated. Finally, as a sensitivity analysis, the fully adjusted models were run on a subset of codes within each violence subtypes (e.g., any burn code for IPV) to examine if certain codes were driving these associations (Additional file 1: Appendix Table 2). There was no null hypothesis significance testing conducted and results instead focus on estimation (Lash 2017). This study was deemed not human research by the University of Minnesota Institutional Review Board.

**Table 2:** Mean violence incidence rate ratios and percent county population characteristics for Minnesota 2004–2014

Violence subtypes	Mean IRR per year (SD)	
	Explicit	Proxy
Child abuse	21 (70)	8 (22)
Elder abuse	2 (5)	106 (196)
Intimate partner violence	5 (19)	294 (762)

Level socio-demographic characteristics	Percent (SD)
Percentage of all ages in poverty	11 (3)
Percentage of people of color^a^	9 (8)
Percentage of unemployed	6 (2)
Percentage less than high school education	17 (7)
Urban n (%)	31 (36)

*IRR* incidence rate ratio

^a^People of color includes people who are American Indian, Asian, Black, Two or more races, and people who are Hispanic of any race

## Results

Table 2 describes the average rates of explicit- and proxy-identified violence subtypes and socio-demographic characteristics across counties in the sample. Rates estimated using explicit and proxy codes were substantially different, especially for elder abuse (2 per 1000 for explicit vs. 106 per 1000 for proxy) and intimate partner violence (5 per 1000 for explicit vs. 294 per 1000 for proxy).

Table 3 describes the crude bivariate associations of year and each county socio-demographic factor with explicit-, proxy-, and combined-identified violence rates. Generally, there is a stronger magnitude of association with county level factors for explicit codes compared to proxy coded.

**Table 3:** Crude bivariate negative binomal regression with GEE: incidence rate ratio for the association between each county level socio-demographic characteristics and injury codes

	Child maltreatment						Elder						IPV					
	Explicit		Proxy		Combined		Explicit		Proxy		Combined		Explicit		Proxy		Combined	
	IRR	95% CI	IRR	95% CI	IRR	95% CI	IRR	95% CI	IRR	95% CI	RR	95% CI	IRR	95% CI	IRR	95% CI	IRR	95% CI
*Year*																		
Year	0.99	0.98–1.00	1.03	1.02–1.04	1.00	0.99–1.01	1.03	1.01–1.06	1.13	1.12–1.14	1.13	1.12–1.13	0.98	0.96–1.01	1.04	1.03–1.04	1.04	1.03–1.04
*Percent of all ages in poverty*																		
Less than 11.3%	Ref	–	Ref	–	Ref	–	Ref	–	Ref	–	Ref	–	Ref	–	Ref	–	Ref	–
11.3% or higher	1.71	1.37–2.13	1.24	0.94–1.62	1.54	1.24–1.91	1.24	0.92–1.68	1.00	0.86–1.16	1.00	0.87–1.16	1.08	0.77–1.53	1.12	0.99–1.26	1.12	0.99–1.27
*Percent of people of color*^a^																		
Less than 9.4%	Ref	–	Ref	–	Ref	–	Ref	–	Ref	–	Ref	–	Ref	–	Ref	–	Ref	–
9.4% or higher	1.57	1.21–2.04	1.35	1.03–1.76	1.50	1.19–1.90	1.55	1.17–2.06	0.96	0.82–1.11	0.96	0.83–1.11	1.31	0.93–1.83	1.17	1.03–1.32	1.17	1.04–1.33
*Percent unemployed*																		
Less than 5.8%	Ref	–	Ref	–	Ref	–	Ref	–	Ref	–	Ref	–	Ref	–	Ref	–	Ref	–
5.8% or higher	1.66	1.31–2.10	1.24	0.94–1.63	1.51	1.21–1.89	1.75	1.34–2.29	1.03	0.89–1.19	1.04	0.90–1.20	1.62	1.19–2.20	1.16	1.03–1.31	1.17	1.04–1.32
*Percent less than high school education*																		
Less than 17.5%	Ref	–	Ref	–	Ref	–	Ref	–	Ref	–	Ref	–	Ref	–	Ref	–	Ref	–
17.5% or greater	1.14	0.87–1.50	0.96	0.74–1.25	1.09	0.85–1.39	0.66	0.51–0.86	1.23	1.07–1.42	1.23	1.07–1.41	0.83	0.60–1.14	1.10	0.98–1.24	1.10	0.98–1.24
*Urban*																		
Rural	Ref	–	Ref	–	Ref	–	Ref	–	Ref	–	Ref	–	Ref	–	Ref	–	Ref	–
Urban	1.00	0.76–1.31	1.01	0.76–1.33	1.00	0.79–1.28	1.36	1.01–1.83	0.84	0.72–0.97	0.84	0.72–0.98	1.11	0.79–1.56	0.95	0.84–1.07	0.95	0.84–1.07

*IRR* incidence rate ratio

^a^People of color includes people who are American Indian, Asian, Black, Two or more races, and people who are Hispanic of any race

The fully adjusted models are in Table 4. Using explicit codes, the rate of elder abuse appears to slightly increase from 2004 to 2014 (IRR_explicit_ per year: 1.03; 95% CI 1.01–1.06). The time trend for child abuse and IPV are both flat or slightly decreasing (child maltreatment IRR_explicit_ per year: 0.98; 95% CI 0.97–1.00, and IPV IRR_explicit_ per year: 0.98; 95% CI 0.96–1.01, respectively). In contrast, using proxy codes, there appears to be a substantial upward trend in elder abuse rates (IRR_proxy_ per year:1.12; 95% CI 1.11–1.13) from 2004 to 2014. Child abuse and IPV measured using proxy codes are slightly increasing over time (child abuse IRR_proxy_ per year :1.03; 95% CI 1.02–1.05, and IPV IRR_proxy_ per year: 1.04; 95% CI 1.03–1.04). The combined explicit and proxy codes for child abuse, elder abuse and intimate partner violence mimic the proxy codes in magnitude and pattern.

**Table 4:** Fully adjusted^b^ negative binomal regression with GEE: incidence rate ratio for the association between all county level socio-demographic characteristics and injury codes

	Child Maltreatment						Elder						IPV					
	Explicit		Proxy		Combined		Explicit		Proxy		Combined		Explicit		Proxy		Combined	
	IRR	95% CI	IRR	95% CI	IRR	95% CI	IRR	95% CI	IRR	95% CI	IRR	95% CI	IRR	95% CI	IRR	95% CI	IRR	95% CI
*Percent of all ages in poverty*																		
Less than 11.3%	Ref	–	Ref	–	Ref	–	Ref	–	Ref	–	Ref	–	Ref	–	Ref	–	Ref	–
11.3% or higher	1.36	1.09–1.68	1.12	0.88–1.43	1.27	1.03–1.55	1.06	0.83–1.36	0.98	0.82–1.16	0.97	0.82–1.15	0.84	0.61–1.16	1.02	0.90–1.16	1.02	0.90–1.16
*Percent of people of color*^a^																		
Less than 9.4%	Ref	–	Ref	–	Ref	–	Ref	–	Ref	–	Ref	–	Ref	–	Ref	–	Ref	–
9.4% or higher	1.46	1.17–1.81	1.37	1.05–1.79	1.43	1.16–1.77	1.42	1.14–1.77	0.98	0.84–1.14	0.98	0.84–1.14	1.30	0.93–1.84	1.16	1.02–1.32	1.16	1.03–1.32
*Percent unemployed*																		
Less than 5.8%	Ref	–	Ref	–	Ref	–	Ref	–	Ref	–	Ref	–	Ref	–	Ref	–	Ref	–
5.8% or higher	1.42	1.17–1.74	1.15	0.88–1.50	1.32	1.08–1.60	1.58	1.27–1.97	1.06	0.90–1.25	1.06	0.91–1.25	1.73	1.30–2.30	1.13	1.00–1.28	1.14	1.01–1.29
*Percent less than high school education*																		
Less than 17.5%	Ref	–	Ref	–	Ref	–	Ref	–	Ref	–	Ref	–	Ref	–	Ref	–	Ref	–
17.5% or greater	1.09	0.90–1.32	0.90	0.67–1.21	1.02	0.84–1.24	0.74	0.58–0.95	1.18	1.00–1.39	1.18	1.00–1.38	0.86	0.64–1.17	1.06	0.94–1.19	1.06	0.94–1.19
*Urban*																		
Rural	Ref	–	Ref	–	Ref	–	Ref	–	Ref	–	Ref	–	Ref	–	Ref	–	Ref	–
Urban	0.95	0.78–1.16	0.88	0.65–1.18	0.92	0.75–1.12	1.03	0.79–1.35	0.91	0.76–1.10	0.91	0.76–1.10	0.99	0.70–1.39	0.93	0.81–1.07	0.93	0.81–1.07
*Year*																		
Year	0.98	0.97–1.00	1.03	1.02–1.05	1.00	0.99–1.01	1.03	1.01–1.06	1.12	1.11–1.13	1.12	1.11–1.13	0.98	0.96–1.01	1.04	1.03–1.04	1.04	1.03–1.04

*IRR* incidence rate ratio

^a^People of color includes people who are American Indian, Asian, Black, Two or more races, and people who are Hispanic of any race

^b^Percent poverty, percent minority, urbanicity, percent unemployed and percent less than high school education

In the fully adjusted models, explicit codes for child abuse indicate that counties with greater than or equal to the mean (11.3%) of people living in poverty have 1.36 (95% CI 1.09–1.68) times the rate of child abuse compared to counties that had less than 11.3% of people living in poverty. In general, the association of child abuse with county-level measures of poverty, people of color, unemployment, and education all decrease in magnitude when comparing explicit to proxy codes, although confidence intervals are often overlapping. For example, using the proxy measure of child abuse, counties with greater than or equal to 11.3% of people living in poverty have 1.12 (95% CI 0.88–1.43) times the rate of child abuse compared to counties with less than 11.3% of people living in poverty. Using combined explicit and proxy codes, counties with more than the average poverty have 1.27 (95% CI 1.03–1.55) times of the rate of child abuse as counties with less than the average poverty. The combined codes tend to be in the middle between proxy and explicit codes.

Using explicit elder abuse codes, counties with greater than or equal to the mean (5.8%) of people unemployed have a 58% increased rate of elder abuse compared to counties that had less than 5.8% unemployed. The associations between elder abuse and county-level socio-demographic characteristics also are lower in magnitude when using proxy rather than explicit codes, except for county-level education. For example, using proxy-identified and combined-identified elder abuse, counties with higher unemployment had about a 6% increased rate of elder abuse compared with counties that had lower unemployment (IRR_proxy_ 1.06, 95% CI 0.90–1.25; IRR_combined_ 1.06, 95% CI 0.91–1.25), respectively.

As with child maltreatment and elder abuse, generally the associations between IPV and county level socio-demographic characteristics were closer to the null, although two associations flipped directionality, when using proxy and combined codes compared with explicit codes. For example, the counties with higher percent unemployment, and those with more people of color, were found to have a greater relative rates of IPV using the explicit versus proxy and combined codes (IRR_explicit_ 1.73, 95% CI 1.30–2.30 vs. IRR_proxy_ 1.13, 95% CI 1.00–1.28 vs. IRR_combined_ 1.14, 95% CI 1.01–1.29) and (IRR_explicit_ 1.30, 95% CI 0.93–1.84 vs. IRR_proxy_ 1.16, 95% CI 1.02–1.32 versus IRR_combined_ 1.16, 95% CI 1.03–1.32), respectively. Associations between IPV and poverty, education and urbanicity were close to the null in regardless of explicit, proxy or combined codes.

Lastly, the sensitivity analysis showed that, in the fully adjusted models, the malnutrition subset codes drove most of the associations with elder abuse and each county level sociodemographic characteristics. The associations between county level sociodemographic characteristics with child abuse and IPV subsets codes were less clear on which individual code drove these associations.

## Discussion

The goal of this paper is to determine how the addition of proxy codes in relation and in combination to a more traditional approach (explicit codes) describe violence using population-based data for one state. Our main findings are that the magnitude of violence rates, and patterns of violence across time and by county-level violence differed depending on whether one used explicit or proxy codes. In particular, explicit codes suggested that child abuse and IPV trends were flat or decreased slightly from 2004 to 2014, while proxy codes suggested the opposite. Elder abuse increased during this timeframe for both explicit and proxy codes, but more dramatically when using proxy codes. In regard to the associations between county level characteristics and each violence subtype, previously identified county-level risk factors were more strongly related to explicitly-identified violence than to proxy-identified violence. Given the larger number of proxy-identified than explicit-identified violence cases, the trends and associations of combined codes align more closely with proxy codes, especially with elder abuse and IPV.

The finding of increasing violence over time using proxy codes contrast with evidence of declining trends of child abuse (Finkelhor et al. 2014), elder abuse (Morgan and Mason 2014) and IPV (Catalano 2013) from data sources such as the Uniform Crime Reports (UCR), Child Protection (Finkelhor et al. 2013a), and the National Crime Victimization Survey (NCVS) (Powers and Kaukinen 2012; Morgan and Kena 2017). There are several possible reasons for differences in findings between this study and these other data sources. Traditional surveillance systems for violence may have systemic selection bias. For example, UCR relies on police data that may be likely to over-report crime in communities of color and under-report in white communities (Myers 1980; Mesic et al. 2018; Voigt et al. 2017). Further, UCR excludes sexual assault, and crimes not reported to the police (Planty et al. 2014). Underreporting is also a problem in Child Protection data (Wildeman et al. 2014). For example, in 2011, the National Child Abuse and Neglect Data System (NCANDS) reported approximately three million U.S. children who received an investigation or response from a state child protection service agency (Maltreatment 2020) but in the same year, according to the National Survey of Children’s Exposure to Violence reported, approximately 10 million U.S. youth had experienced maltreatment by their caregiver (Finkelhor et al. 2013b). Therefore, different types of selection and usage of these different surveillance systems (such as health care utilization) could be a reason behind different trends because each system is measuring different populations or is measuring violence differently.

Generally, the associations between violence and county socio-demographic compositional factors are smaller for proxy codes than for explicit codes. For example, after adjustment for all other county-level demographic characteristics, explicit codes indicated that violent injuries for elder abuse are highest in counties that had population percentages at or above the Minnesota mean percent of people of color. When violence is measured with proxy codes or combined codes, these associations are still elevated but move toward the null. One possible explanation of this could be that proxy codes could be less systemically racially biased, or conversely, explicit codes are more greatly influenced by racial bias. Explicit codes require a subjective judgment by medical providers, which leaves them vulnerable to individuals’ implicit biases. Proxy codes do not require this judgment and may be less affected by this bias. On the other hand, proxy codes trade greater potential representativeness for lower specificity, potentially leading to non-differential misclassification, which could also move effect estimates closer to the null. These different associations by proxy versus explicit coding suggest that sole reliance on explicit coding of violence for surveillance and research may be insufficient and proxy codes may potentially help to address under- and biased reporting (Shepherd and Sivarajasingam 2005), yet research is required to understand the potential misclassification in proxy codes. Since ICD codes are a universal coding system in the U.S., further testing and application should be done to assess proxy codes’ validity for violence identification.

The differences between predictors and trends over time for proxy and explicit codes is unknown. The differences, in part, are likely due to a trade off in specificity and sensitivity of the codes. For instance, explicit codes likely have high specificity (i.e. those with violence codes are very likely to reflect true violence), but may suffer from low sensitivity (i.e. many/most cases of violence are not coded as violence, and thus miss many cases of violence). If this misclassification is non-differential (Aschengrau and Seage 2021) with respect to predictor variables, then it may attenuate associations. However, if, as suspected, this misclassification is differential due to greater suspicion of violence in certain populations associated with our hardship measures, then associations may be biased away from the null. Proxy codes could see a decrease in specificity but an increase in sensitivity and the final estimate may still be an underestimate of the actual effect but perhaps closer in magnitude to the actual effect. These codes are also misclassified, but perhaps in a less systematic way (less biased). The use of proxy codes allows for violence cases to be identified that may not have been otherwise detected which is important for prevention. The utility of proxy codes for prevention may make these codes more forgiving of false positives. To our knowledge, this is the first study to compare the predicters and trends over time for proxy and explicit codes, therefore, future validation work is important.

This study has limitations that should be considered. First, this study missed those who experienced violent events but did not go to the hospital. Second, hospital data may be an oversample of those with health insurance in the population. That said, more severe or urgent injuries may bring people in for care despite the lack of health insurance coverage (Sommers and Simon 2017). Thus, this study may be seen as an analysis of more severe violence-related injuries. Third, hospital data lack details such as perpetration and location of the event that are available in studies like UCR, NCVS, and NCANDS, which could help to identity potential points for intervention. Fourth, the analysis includes a set of county-level covariates from a single time point (the 2010 decennial census or ACS). This limits the ability to account for variation across time in the covariates. However, there is minimal change over this 11-year time frame in these measures at the state level (Minnesota Compass 2020), and a middle timepoint captures mean covariate levels across the study period. Fifth, while these data are representative of violent related injuries in Minnesota given the census of hospital records as the data source, the results may not be generalizable outside of Minnesota. Sixth, this study uses ICD-9 codes while the current version of hospital discharge codes is ICD-10, thus the study stopped at 2014. Despite the use of an older coding system, both injury codes (ICD-9 and ICD-10) continue to use a similar approaches that are translatable (Gibson et al. 2016). In addition, the ICD-10 external cause framework is developed to be as consistent as possible with ICD-9 codes (Injury Data and Resources 2019).

This study has several strengths that mitigate its weaknesses. First, this study utilizes a population-based administrative dataset at the county level for Minnesota, allowing generalizability to the entire population. Second, while proxy codes are likely to have some misclassification, they are subject to potentially less systemic bias and may be thus better capture violence in communities where violence is not traditionally identified, such as whiter or wealthier communities (Sumner et al. 2015). Given the different strengths and weaknesses of explicit and proxy codes, and the lack of a gold standard for violence identification, it is useful to consider both approaches in research and for replication in other studies. The combined code approach could be a possible way to be more inclusive for studies that are attempting to target a broad pool. Third, this study utilizes county-level geography to distinguish associations between violence and county socio-demographic characteristics, which can be useful for local public health agency surveillance. In contrast, violence trend data such as NCVS are commonly reported at the nation or region level, which limits the more granular assessment of trends that occur in different parts of the United States.

There are several implications for future research from these findings. Violence surveillance utilizing hospital discharge data, and particularly proxy codes, may add important data that traditional surveillance lacks. Most importantly, explicit and proxy codes indicate different geographic patterns and trends over time. The use of proxy codes for violence identification may provide an avenue for capturing violence that traditional surveillance misses (Boyle and Kirkbride 2005). Accurate surveillance of violence is critical for resource allocation for prevention and intervention. Utilizing proxy codes in conjunction with explicit codes may be one step towards more comprehensive surveillance. More specifically, hospital records could be used as a syndromic surveillance system for violence, which could lead to potentially more timely and impactful interventions. Future research examining hospital discharge data for violence identification utilizing and verifying proxy codes can help to identify the hidden burden of violence.

## Supplementary Information

**Additional file 1. Appendix Table S1:** Proxy Codes used to Define Violence. **Appendix Table S2:** Fully Adjusted^b^ Negative Binomal Regression with GEE: Rate Ratio for the Association Between All County Level Socio-demographic Characteristics and Subtypes of Proxy Injury Codes.

## Data Availability

The majority of the data that support the findings of this study are available from Minnesota Hospital Association but restrictions apply to the availability of these data, which were used under license for the current study, and so are not publicly available. The remaining data from the American Community Survey are available from the United States Census Bureau, website https://www.census.gov/programs-surveys/acs.

## Abbreviations

ICD-9 CM codes: International classification of disease clinical modification, 9th revision
CPS: Child protective services
E-codes: External cause
IPV: Intimate partner violence
APS: Adult protective services
MHA: Minnesota hospital association
NCVS: National crime victimization survey
UCR: Uniform crime reports
NCANDS: National child abuse and neglect data system
ACS: American community survey
SEER: Surveillance, epidemiology, and end results

## References

[CR1] Ahern J, Matthay EC, Goin DE, Farkas K, Rudolph KE (2018). Acute changes in community violence and increases in hospital visits and deaths from stress-responsive diseases. Epidemiology.

[CR2] Aschengrau A, Seage GR. Essentials of epidemiology in public health, 4th edn. Independently Published; 2021. 538 p.

[CR3] Barlow KM, Milne S, Aitken K, Minns RA (1998). A retrospective epidemiological analysis of non-accidental head injury in children in Scotland over a 15 year period. Scott Med J.

[CR4] Beynon CE, Gutmanis IA, Tutty LM, Wathen N, Macmillan HL (2012). Why physicians and nurses ask (or don’t) about partner violence: a qualitative analysis. BMC Public Health.

[CR5] Bhandari M, Dosanjh S, Tornetta P, Matthews D (2006). Musculoskeletal manifestations of physical abuse after intimate partner violence. J Trauma Injury Infect Crit Care.

[CR6] Bhargava R, Temkin TL, Fireman BH, Eaton A, Mccaw BR, Kotz KJ (2011). A predictive model to help identify intimate partner violence based on diagnoses and phone calls. AMEPRE.

[CR7] Boyle A, Kirkbride J (2005). Record linkage of domestic assault victims between an emergency department and the police. J Epidemiol Community Health.

[CR8] Btoush R, Campbell JC, Gebbie KM (2009). Care provided in visits coded for intimate partner violence in a national survey of emergency departments. Women’s Health Issues.

[CR9] Catalano S. Intimate partner violence: attributes of victimization, 1993–2011. 2013.

[CR10] Child Maltreatment. Children’s Bureau | ACF [Internet]. 2020. [cited 2020 Feb 11]. Available from: https://www.acf.hhs.gov/cb/research-data-technology/statistics-research/child-maltreatment.

[CR11] Davidov DM, Larrabee H, Davis SM (2015). United States emergency department visits coded for intimate partner violence. J Emerg Med.

[CR12] Edwards KM (2015). Intimate partner violence and the rural–urban–suburban divide. Trauma Violence Abuse.

[CR13] Feldman J, Gruskin S, Coull B, Krieger N (2017). Quantifying underreporting of law-enforcement-related deaths in United States vital statistics and news-media-based data sources: a capture–recapture analysis. PLOS Med.

[CR14] Finkelhor D, Jones LM, Shattuck AM, Finkelhor D, Jones L, Shattuck A, et al. Updated trends in child maltreatment, 2012. Durham, NH; 2013a.

[CR15] Finkelhor D, Turner HA, Shattuck A, Hamby SL (2013). Violence, crime, and abuse exposure in a national sample of children and youth an update. JAMA Pediatr.

[CR16] Finkelhor D, Shattuck A, Turner HA, Hamby SL (2014). Trends in children’s exposure to violence, 2003 to 2011. JAMA Pediatr.

[CR17] Gibson T, Casto A, Young J, Karnell L, Coenen N. Impact of ICD-10-CM/PCS on Research using administrative databases [Internet]. U.S. Agency for Healthcare Research and Quality; 2016. (HCUP Methods Series Report # 2016-02). Available from: http://www.hcup-us.ahrq.gov/reports/methods/methods.jsp.

[CR18] Gironda MW, Nguyen AL, Mosqueda LM (2016). Is this broken bone because of abuse? Characteristics and comorbid diagnoses in older adults with fractures. J Am Geriatr Soc.

[CR19] Gray BJ, Barton ER, Davies AR, Long SJ, Roderick J, Bellis MA (2017). A shared data approach more accurately represents the rates and patterns of violence with injury assaults. J Epidemiol Community Health.

[CR20] Halpern LR, Parry BA, Hayward G, Peak D, Dodson TB (2009). A comparison of 2 protocols to detect intimate partner violence. J Oral Maxillofac Surg.

[CR21] Hooft AM, Asnes AG, Livingston N, Deutsch S, Cahill L, Wood JN (2015). The accuracy of ICD codes: identifying physical abuse in 4 Children’s hospitals. Acad Pediatr.

[CR22] Hymel KP, Laskey AL, Crowell KR, Wang M, Armijo-garcia V, Frazier TN (2018). Racial and ethnic disparities and bias in the evaluation and reporting of abusive head trauma. J Pediatr.

[CR23] Injury Data and Resources. ICD Injury Matrices [Internet]. Centers for Disease Control and Prevention. 2015 [cited 2020 Mar 11]. Available from: https://www.cdc.gov/nchs/injury/injury_matrices.htm.

[CR24] Injury Data and Resources. ICD Injury Matrices [Internet]. 2019. [cited 2021 Jan 30]. Available from: https://www.cdc.gov/nchs/injury/injury_matrices.htm

[CR25] Keenan HT, Campbell KA, Page K, Cook LJ, Bardsley T, Olson LM (2017). Perceived social risk in medical decision-making for physical child abuse: a mixed-methods study. BMC Pediatr.

[CR26] Lachs MS, Williams CS, O’Brien S, Hurst L, Kossack A, Siegal A (1997). ED use by older victims of family violence. Ann Emerg Med.

[CR27] Lane WG, Rubin DM, Monteith R, Christian CW (2002). Racial differences in the evaluation for physical abuse. JAMA.

[CR28] Lash TL (2017). The harm done to reproducibility by the culture of null hypothesis significance testing. Am J Epidemiol.

[CR29] Lipsky S, Caetano R, Roy-byrne P (2009). Racial and ethinic disparities in police-reported intimate partner violence and risk of hospitalization among women. Women’s Health Issues.

[CR30] Lipsky S, Cristofalo M, Reed S, Caetano R, Roy-Byrne P (2012). Racial and ethnic disparities in police-reported intimate partner violence perpetration: a mixed methods approach. J Interpers Violence.

[CR31] Lloyd SS, Rissing JP (1985). Physician and Coding errors in patient records. JAMA J Am Med Assoc.

[CR32] Maguire-Jack K, Cao Y, Yoon S (2018). Racial disparities in child maltreatment: the role of social service availability. Child Youth Serv Rev.

[CR33] Minnesota Compass. Demographics : overview [Internet]. Wilder Research. 2020. [cited 2020 Mar 12]. Available from: http://www.mncompass.org/demographics/overview.

[CR34] McKenzie K, Scott DA, Waller GS, Campbell M (2011). Reliability of routinely collected hospital data for child maltreatment surveillance. BMC Public Health.

[CR35] Mesic A, Franklin L, Cansever A, Potter F, Sharma A, Knopov A (2018). The relationship between structural racism and black–white disparities in fatal police shootings at the state level. J Natl Med Assoc.

[CR36] Morgan RE, Jennifer TL. Criminal victimization, 2019. 2020. p. 53. Report No.: NCJ 255113.

[CR37] Morgan R, Kena G (2017). Criminal victimization, 2016. Bureau Justice Stat.

[CR38] Morgan RE, Mason BJ. Crimes against the elderly, 2003–2013. 2014.

[CR39] Muldoon K, Smith G, Talarico R, Heimerl M, McLean C, Sampsel K (2019). A 15-year population-based investigation of sexual assault cases across the province of Ontario, Canada, 2002–2016. Am J Public Health.

[CR40] Myers SL (1980). Why are crimes underreported? What is the crime rate? Does it really matter?. Soc Sci Q.

[CR41] Nannini A, Lazar J, Berg C, Barger M, Tomashek K, Cabral H (2008). Physical injuries reported on hospital visits for assault during the pregnancy-associated period. Nurs Res.

[CR42] National Cancer Institute. SEER Data & Software [Internet]. 2021. [cited 2021 Mar 1]. Available from: https://seer.cancer.gov/data-software/index.html.

[CR43] Perciaccante VJ, Ochs HA, Dodson TB (1999). Head, neck, and facial injuries as markers of domestic violence in women. J Oral Maxillofac Surg.

[CR44] Perciaccante VJ, Carey JW, Susarla SM, Dodson TB (2010). Markers for intimate partner violence in the emergency department setting. J Oral Maxillofac Surg.

[CR45] Petridou E, Browne A, Lichter E, Dedoukou X, Alexe D, Dessypris N (2002). What distinguishes unintentional injuries from injuries due to intimate partner violence: a study in Greek ambulatory care settings. Injury Prev.

[CR46] Planty M, Langston L, Barnett-Ryan C. The nation’s two crime measures. The Bureau of Justice Statistics of the US Department of Justice. 2014.

[CR47] Powers RA, Kaukinen CE (2012). Trends in intimate partner violence: 1980–2008. J Interpers Violence.

[CR48] Reis BY, Kohane IS, Mandl KD (2009). Longitudinal histories as predictors of future diagnoses of domestic abuse: modelling study. BMJ.

[CR49] Rochelle S, Buonanno L (2018). Charting the attitudes of county child protection staff in a post-crisis environment. Child Youth Serv Rev.

[CR50] Rosen T, Clark S, Bloemen EM, Mulcare MR, Stern ME, Hall JE (2016). Geriatric assault victims treated at U.S. trauma centers: five-year analysis of the national trauma data bank. Injury.

[CR51] Schafer SD, Drach LL, Hedberg K, Kohn MA (2008). Using diagnostic codes to screen for intimate partner violence in oregon emergency departments and hospitals. Public Health Rep.

[CR52] Schnitzer PG, Slusher PL, Kruse RL, Tarleton MM (2011). Identification of ICD codes suggestive of child maltreatment. Child Abuse Negl.

[CR53] Scott D, Tonmyr L, Fraser J, Walker S, McKenzie K (2009). The utility and challenges of using ICD codes in child maltreatment research: a review of existing literature. Child Abuse Neglect.

[CR54] Shepherd J, Sivarajasingam V (2005). Injury research explains conflicting violence trends. Injury Prev.

[CR55] Sheridan DJ, Nash KR (2007). Acute injury patterns of intimate partner violence victims. Trauma Violence Abuse.

[CR56] Sommers BD, Simon K (2017). Health insurance and emergency department use—a complex relationship. N Engl J Med.

[CR57] Sumner SA, Mercy JA, Dahlberg LL, Hillis SD, Klevens J, Houry D (2015). Violence in the United States: status, challenges, and opportunities. JAMA J Am Med Assoc.

[CR58] Thomas SK, Brooks SE, Mullins CD, Baquet CR, Merchant S (2002). Use of ICD-9 coding as a proxy for stage of disease in lung cancer. Pharmacoepidemiol Drug Saf.

[CR59] United States Census Bureau (2020) When to Use 1-year, 3-year, or 5-year Estimates [Internet]. 2019 [cited 2020 Jun 30]. Available from: https://www.census.gov/programs-surveys/acs/guidance/estimates.html.

[CR60] US Census Bureau. Decennial Census by Decades [Internet]. The United States Census Bureau. 2021a. [cited 2021 Sep 6]. Available from: https://www.census.gov/programs-surveys/decennial-census/decade.html.

[CR61] US Census Bureau. American Community Survey (ACS) [Internet]. The United States Census Bureau. 2021b. [cited 2021 May 4]. Available from: https://www.census.gov/programs-surveys/acs.

[CR62] Voigt R, Camp NP, Prabhakaran V, Hamilton WL, Hetey RC, Griffiths CM (2017). Language from police body camera footage shows racial disparities in officer respect. Proc Natl Acad Sci USA.

[CR63] Wiglesworth A, Austin R, Corona M, Mosqueda L (2009). Bruising as a marker of physical elder abuse: bruising as a marker of physical elder abuse. J Am Geriatr Soc.

[CR64] Wildeman C, Emanuel N, Leventhal JM, Putnam-Hornstein E, Waldfogel J, Lee H (2014). The prevalence of confirmed maltreatment among US children, 2004 to 2011. JAMA Pediatr.

[CR65] Wu V, Huff H, Bhandari M (2010). Pattern of physical injury associated with intimate partner violence in women presenting to the emergency department: a systematic review and meta-analysis. Trauma Violence Abuse.

