# Supplementary material for: Measuring the hidden burden of violence: use of explicit and proxy codes in Minnesota injury hospitalizations, 2004–2014
Source: Inj Epidemiol. 2021 Nov 1;8:63. doi: 10.1186/s40621-021-00354-6 (PMC8559360; doi:10.1186/s40621-021-00354-6)

| **Additional file 1: Appendix Table S1. Proxy Codes used to Define Violence** | | | | | | | | | | | | |
| --- | --- | --- | --- | --- | --- | --- | --- | --- | --- | --- | --- | --- |
|  |  | Code(s) | Age Restriction | n^a^ |  |  | Code(s) | Exclusion Code(s) | Age Restriction | Sex | n^a^ |  |
| Child sexual abuse related codes | |  |  |  | Child neglect or physical abuse related codes | |  |  |  |  |  |  |
|  | Genital herpes | 54.1 | < 10 | 1207 |  | Skull vault fracture | 800 | N/A | < 5 | N/A | 0 |  |
|  | Gonococcal infection | 98 | < 10 | 0 |  | Vertebral fracture | 805 | N/A | < 5 | N/A | 0 |  |
|  | Pelvic inflammatory disease, unspecified | 614.9 | < 10 | 6 |  | Traumatic subarachnoid hemorrhage | 852 | N/A | < 5 | N/A | 0 |  |
|  | Contusion of genital organs | 922.4 | < 10 | 1035 |  | Intrathoracic injury | 862 | N/A | < 5 | N/A | 0 |  |
|  | Observation after alleged rape | V71.5 | < 10 | 2531 |  | Small intestine injury | 863.2,863.3 | N/A | < 5 | N/A | 0 |  |
|  | Observation for abuse/neglect | V71.81 | < 10 | 4370 |  | Spleen injury | 865 | N/A | < 5 | N/A | 0 |  |
| Child neglect related codes | |  |  |  |  | Spinal cord injury | 952 | N/A | < 3 | N/A | 0 |  |
|  | Other severe malnutrition | 262 | <10 | 2 | Child physical abuse related codes | |  |  |  |  |  |  |
|  | Dental caries | 521 | <10 | 0 |  | Retinal hemorrhage | 362.81 | N/A | < 3 | N/A | 44 |  |
|  | Solar radiation dermatitis | 692.7 | < 2 | 0 |  | Rib fracture | 807.0, 807.1 | N/A | < 5 | N/A | 0 |  |
|  | Pelvic fracture | 808 | < 5 | 0 |  | Scapula fracture | 811 | N/A | < 5 | N/A | 0 |  |
|  | Traumatic pneumohemothorax | 860 | < 5 | 0 |  | Traumatic subdural hemorrhage | 852.2 | N/A | < 5 | N/A | 0 |  |
|  | Heart or lung injury | 861 | < 5 | 0 |  | Other/unspecified intracranial hemorrhage | 853 | N/A | < 5 | N/A | 0 |  |
|  | GI injury | 863.8 | < 5 | 0 |  | Stomach injury | 863.1 | N/A | < 10 | N/A | 1 |  |
|  | Liver injury | 864 | < 5 | 0 |  | Assault | E965, E966, E968.2 | N/A | < 4 | N/A | 53 |  |
|  | Kidney injury | 866 | < 5 | 0 |  | Assault, NOS | E968.9 | N/A | < 4 | N/A | 97 |  |
|  | Burn of head | 941 | < 5 | 0 |  | Undetermined intent, other means | E988 | N/A | < 10 | N/A | 0 |  |
|  | Burn of trunk | 942 | < 5 | 0 | Intimate partner violence | |  |  |  |  |  |  |
|  | Burn of leg | 945 | < 5 | 0 |  | Any burn injury to the face/head | 940.0- 940.5, 940.9, 941.00-941.59 | E810.0-E819.9 | >=16 | Female | 5511 |  |
|  | Burn of multiple sites | 946 | < 5 | 0 |  | Any open wound injury to the face/head | 873.0-873.9, 910(.0-.3,.6-.9), 918.9, 959.01, 959.09 | E810.0-E819.9 | >=16 | Female | 330910 |  |
|  | Poisoning by drugs/medicinals | 960–979 | < 5 | 0 |  | Fracture of face bones | 802.0-802.9 | E810.0-E819.9 | >=16 | Female | 16078 |  |
|  | Drowning, non-fatal submersion | 994.1 | < 4 | 415 |  | Contusion to face/scalp, and neck | 920.0-921.9 | E810.0-E819.9 | >=16 | Female | 113848 |  |
|  | Second-hand tobacco smoke | E869.4 | <10 | 1606 |  | Any vessel injury to the face/head | 900.0-900.9 | E810.0-E819.9 | >=16 | Female | 96 |  |
|  | Swimming accident | E910.2 | < 4 | 68 | Elder Abuse | |  |  |  |  |  |  |
|  | Bathtub (near) drowning | E910.4 | < 4 | 247 |  | Abrasion | 910-919 | E928.9 | >=65 | N/A | 0 |  |
|  | Other (near) drowning | E910.8 | < 4 | 355 |  | Bruise | 920–924 | E928.9 | >=65 | N/A | 47331 |  |
|  | Accidental (near) drowning, | E910.9 | < 4 | 46 |  | Burns | 940–949 | E928.9 | >=65 | N/A | 0 |  |
|  | Unarmed fight, brawl | E960.0 | < 4 | 98 |  | Dehydration | 276.51 | E928.9 | >=65 | N/A | 91478 |  |
|  | Undetermined intent, poisoning | E980 | < 5 | 0 |  | Laceration | 870–897 | E928.9 | >=65 | N/A | 0 |  |
|  | Undetermined intent, firearm | E985 | <10 | 0 |  | Malnutrition | 262–263 | E928.9 | >=65 | N/A | 110 |  |
|  | Household circumstances | V60 | <10 | 0 |  | Pressure ulcer | 707 | E928.9 | >=65 | N/A | 0 |  |
|  |  |  |  |  |  | Strangulation | E963 | E928.9 | >=65 | N/A | 9 |  |
| ^a^Total count. Does not sum to sample size because there could be multiple diagnoses per individual. | | | | | | | | | | | | |

| **Additional file 1: Appendix Table S2: Fully Adjusted^b^ Negative Binomal Regression with GEE: Rate Ratio for the Association Between All County Level Socio-demographic Characteristics and Subtypes of Proxy Injury Codes** | | | | | | | | | | | | | | | | | | | | | | |
| --- | --- | --- | --- | --- | --- | --- | --- | --- | --- | --- | --- | --- | --- | --- | --- | --- | --- | --- | --- | --- | --- | --- |
|  | Child Maltreatment | | | | | | Elder Abuse | | | | | | Intimate Partner Violence | | | | | | | | | |
|  | Sexual Abuse Related codes | | Neglect Related codes | | Physical Abuse Related codes | | Bruise | | Dehydration | | Malnutrition | | Burn | | Wound | | Fracture | | Contusion | | Vessel | |
|  | IRR | 95% CI | IRR | 95% CI | IRR | 95% CI | IRR | 95% CI | IRR | 95% CI | IRR | 95% CI | IRR | 95% CI | IRR | 95% CI | IRR | 95% CI | IRR | 95% CI | IRR | 95% CI |
| **Percent of all ages in poverty** | | | |  |  |  |  |  |  |  |  |  |  |  |  |  |  |  |  |  |  |  |
| Less than 11.3 % | Ref | - | Ref | - | Ref | - | Ref | - | Ref | - | Ref | - | Ref | - | Ref | - | Ref | - | Ref | - | Ref | - |
| 11.3% or higher | 1.31 | 1.05-1.64 | 0.79 | 0.47-1.32 | 1.37 | 0.72-2.60 | 0.90 | 0.76-1.06 | 1.04 | 0.84-1.29 | 1.59 | 0.89-2.84 | 1.02 | 0.86-1.21 | 1.02 | 0.90-1.17 | 0.99 | 0.84-1.17 | 1.03 | 0.88-1.20 | 0.89 | 0.52-1.54 |
| **Percent of people of color** | | |  |  |  |  |  |  |  |  |  |  |  |  |  |  |  |  |  |  |  |  |
| Less than 9.4% | Ref | - | Ref | - | Ref | - | Ref | - | Ref | - | Ref | - | Ref | - | Ref | - | Ref | - | Ref | - | Ref | - |
| 9.4% or higher | 1.43 | 1.16-1.75 | 1.28 | 0.63-2.60 | 1.21 | 0.40-3.65 | 1.03 | 0.89-1.20 | 0.93 | 0.76-1.15 | 1.31 | 0.77-2.21 | 1.01 | 0.88-1.17 | 1.19 | 1.05-1.35 | 1.28 | 1.07-1.53 | 1.11 | 0.95-1.29 | 0.96 | 0.55-1.66 |
| **Percent unemployed** | | |  |  |  |  |  |  |  |  |  |  |  |  |  |  |  |  |  |  |  |  |
| Less than 5.8% | Ref | - | Ref | - | Ref | - | Ref | - | Ref | - | Ref | - | Ref | - | Ref | - | Ref | - | Ref | - | Ref | - |
| 5.8% or higher | 1.25 | 1.02-1.53 | 1.10 | 0.63-1.93 | 0.95 | 0.48-1.91 | 1.06 | 0.90-1.25 | 1.05 | 0.86-1.29 | 0.95 | 0.53-1.69 | 1.11 | 0.94-1.31 | 1.11 | 0.98-1.27 | 1.18 | 1.01-1.38 | 1.15 | 0.99-1.33 | 0.96 | 0.55-1.68 |
| **Percent less than high school education** | | |  |  |  |  |  |  |  |  |  |  |  |  |  |  |  |  |  |  |  |  |
| Less than 17.5% | Ref | - | Ref | - | Ref | - | Ref | - | Ref | - | Ref | - | Ref | - | Ref | - | Ref | - | Ref | - | Ref | - |
| 17.5% or greater | 1.02 | 0.83-1.26 | 0.70 | 0.37-1.32 | 1.05 | 0.35-3.20 | 1.11 | 0.96-1.28 | 1.25 | 1.00-1.57 | 0.48 | 0.22-1.07 | 1.19 | 0.99-1.42 | 1.03 | 0.91-1.17 | 1.08 | 0.92-1.26 | 1.13 | 0.98-1.30 | 1.11 | 0.57-2.15 |
| **Urban** |  |  |  |  |  |  |  |  |  |  |  |  |  |  |  |  |  |  |  |  |  |  |
| Rural | Ref | - | Ref | - | Ref | - | Ref | - | Ref | - | Ref | - | Ref | - | Ref | - | Ref | - | Ref | - | Ref | - |
| Urban | 0.95 | 0.77-1.16 | 0.78 | 0.39-1.55 | 0.76 | 0.19-3.12 | 1.13 | 0.96-1.32 | 0.80 | 0.62-1.04 | 0.58 | 0.27-1.21 | 0.89 | 0.74-1.07 | 0.96 | 0.83-1.10 | 0.90 | 0.75-1.08 | 0.93 | 0.79-1.10 | 0.59 | 0.31-1.12 |
| **Year** |  |  |  |  |  |  |  |  |  |  |  |  |  |  |  |  |  |  |  |  |  |  |
| Year | 1.03 | 1.02-1.05 | 1.03 | 1.00-1.07 | 0.58 | 0.44-0.76 | 1.05 | 1.04-1.06 | 1.19 | 1.18-1.20 | 1.03 | 0.93-1.14 | 0.99 | 0.98-1.01 | 1.05 | 1.05-1.06 | 1.03 | 1.02-1.04 | 1.03 | 1.02-1.04 | 0.92 | 0.85-1.00 |
|  |  |  |  |  |  |  |  |  |  |  |  |  |  |  |  |  |  |  |  |  |  |  |
| IRR=Incidence Rate Ratio | | | | | | |  |  |  |  |  |  |  |  |  |  |  |  |  |  |  |  |
| ^a^People of color includes people who are American Indian, Asian, Black, Two or more races, and people who are Hispanic of any race. | | | | | | | | | | | | | | |  |  |  |  |  |  |  |  |
| ^b^Percent poverty, percent minority, urbanicity, percent unemployed and percent less than high school education. | | | | | | | | | | | | |  |  |  |  |  |  |  |  |  |  |


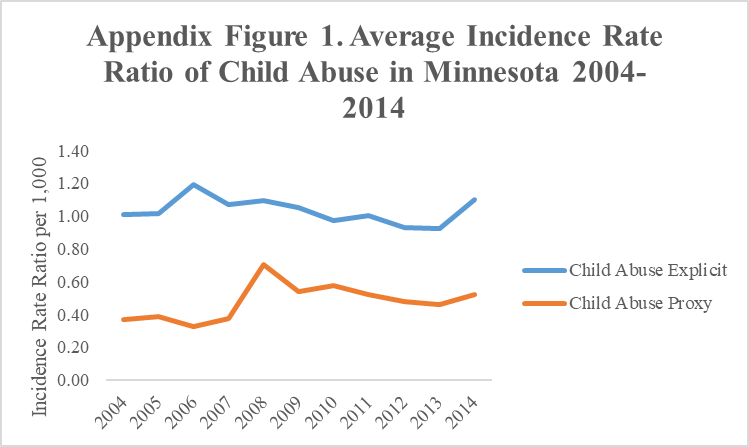

Supplement: Supplementary file 1 — Additional file 1. Appendix Table S1: Proxy Codes used to Define Violence. Appendix Table S2: Fully Adjustedb Negative Binomal Regression with GEE: Rate Ratio for the Association Between All County Level Socio-demographic Characteristics and Subtypes of Proxy Injury Codes. [file 40621_2021_354_MOESM1_ESM.docx]
